# Supplementary material for: The Estonian Biobank’s journey from biobanking to personalized medicine
Source: Nat Commun. 2025 Apr 5;16:3270. doi: 10.1038/s41467-025-58465-3 (PMC11972354; doi:10.1038/s41467-025-58465-3)
Supplement: Supplementary file 1 — Supplementary Info [file 41467_2025_58465_MOESM1_ESM.pdf]

Supplementary Information

From Biobanking to Personalized Medicine: The Journey of the Estonian Biobank

Lili Milani[1,2*], Maris Alver[1], Sven Laur[3,4], Sulev Reisberg[3,4], Toomas Haller[1], Oliver Aasmets[1], Erik Abner[1], Helene Alavere[2], Annely Allik[2], Tarmo Annilo[1], Krista Fischer[1,5], Robin Hofmeister[1,6], Georgi Hudjashov[1,7], Maarja Jõeloo[1], Mart Kals[1], Liis Karo-Astover[1], Silva Kasela[1], Anastassia Kolde[1,5], Kristi Krebs[1], Kertu Liis Krigul[1], Jaanika Kronberg[1], Karoliina Kruusmaa[1], Viktorija Kukuškina[1], Kadri Kõiv[1], Kelli Lehto[1], Liis Leitsalu[1], Sirje Lind[2], Laura Birgit Luitva[1,5], Kristi Läll[1], Kreete Lüll[1], Kristjan Metsalu[2], Mait Metspalu[7], René Mõttus[8], Mari Nelis[1], Tiit Nikopensius[1], Miriam Nurm[1], Margit Nõukas[1], Marek Oja[3], Elin Org[1], Marili Palover[1], Priit Palta[1], Vasili Pankratov[9], Kateryna Pantiukh[1], Natalia Pervjakova[1], Natàlia Pujol-Gualdo[1], Anu Reigo[1], Ene Reimann[1], Steven Smit[2], Diana Sokurova[2], Dage Särg[1], Nele Taba[1], Harry-Anton Talvik[3,4], Maris Teder-Laving[1], Neeme Tõnisson[1], Mariliis Vaht[1], Uku Vainik[1,8], Urmo Võsa[1], Burak Yelmen[1], Tõnu Esko[1], Raivo Kolde[3], Reedik Mägi[1], Jaak Vilo[3,4], Triin Laisk[1], Andres Metspalu[1*]

1 – Estonian Genome Centre, Institute of Genomics, University of Tartu, Estonia

2 – Estonian Biobank, Institute of Genomics, University of Tartu, Estonia

3 – Institute of Computer Science, University of Tartu, Estonia

4 – STACC, Estonia

5 – Institute of Mathematics and Statistics, University of Tartu, Estonia

6 – Department of Computational Biology, University of Lausanne, Lausanne, Switzerland.

7 – Estonian Biocentre, Institute of Genomics, University of Tartu, Estonia

8 – Institute of Psychology, University of Tartu, Estonia

9 – Centre for Genomics, Evolution and Medicine, Institute of Genomics, University of Tartu, Estonia

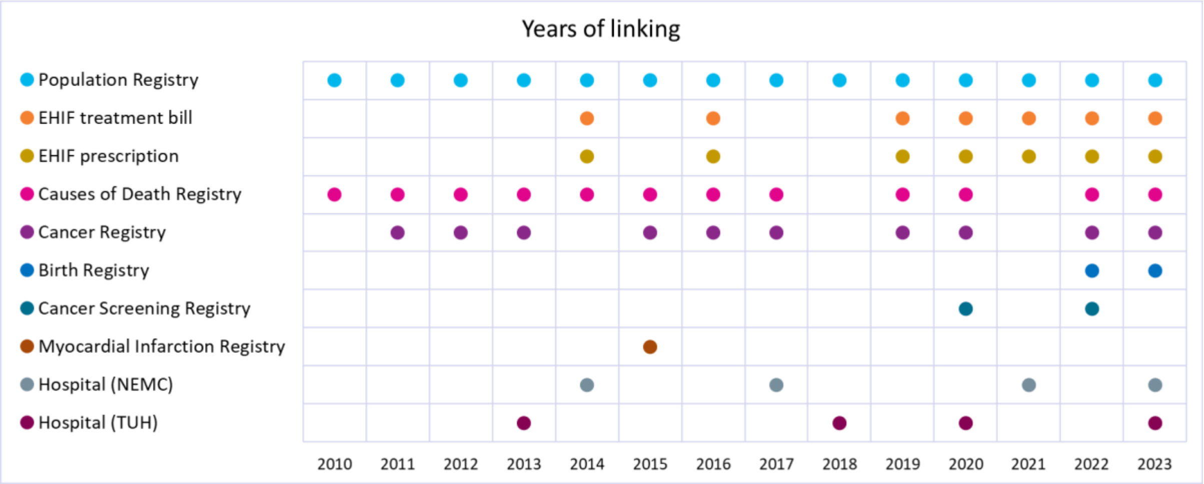

Supplementary Figure 1. Overview of different registries and data sources, and the frequency of data linking in EstBB. Different colored dots represent different sources (see legend on the left). EHIF – Estonian Health Insurance Fund, NEMC – North Estonian Medical Centre, TUH – Tartu University Hospital.

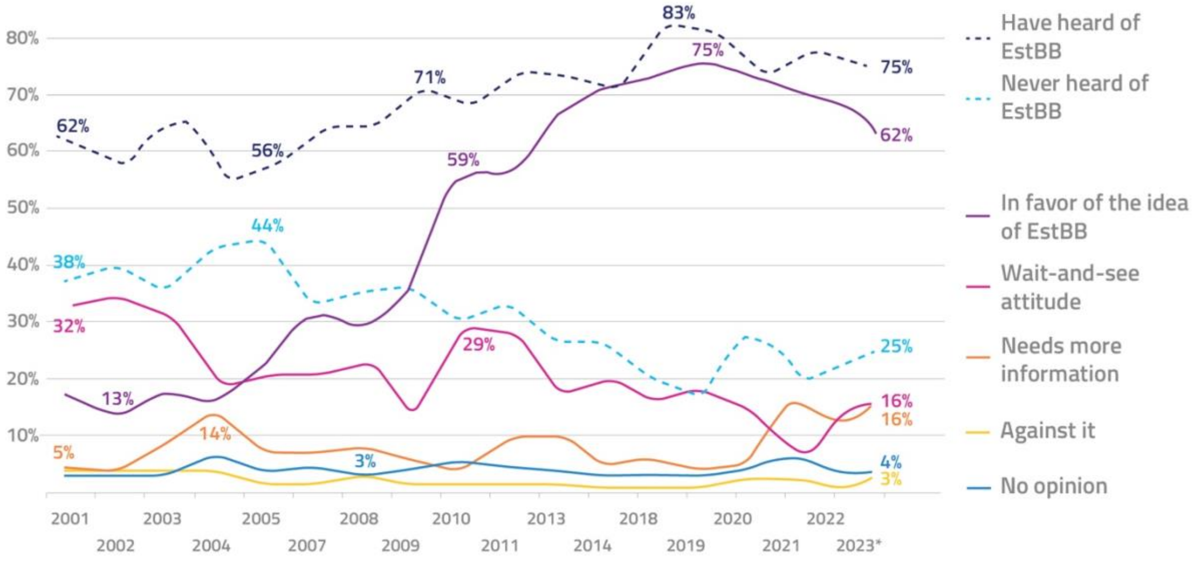

Supplementary Figure 2. Public awareness (dashed line) and attitudes (solid line) towards EstBB in the Estonian population. Each year, ~1000 Estonian residents between the ages of 15 and 74 years were surveyed by a polling agency (TNS Emor). The sample was composed to be proportional to the population structure with respect to age, sex, region, and nationality. *) The 2023 survey was finalized in January 2024.

Supplementary Table 1. Overview of the EstBB questionnaire-based data collections.

Questionnaire	Modules	Year	N of respondents	Questionnaire link
Baseline questionnaire 1	• Sociodemographics<br>• Alcohol, tobacco and other substance use<br>• Physical activity<br>• Anthropometry (weight, height, waist and hip circumference) and objective measurements (blood pressure, pulse rate) *<br>• Sleep<br>• General health<br>• Medical history<br>• Female health<br>• Medical family history<br>• Dietary habits<br>• Medication use and side effects	2004-2010	52,000	
Baseline questionnaire 2	• Sociodemographics<br>• Alcohol, tobacco and other substance use<br>• Physical activity<br>• Anthropometry (weight, height, waist and hip circumference) **<br>• Sleep<br>• General health<br>• Female health<br>• Dietary habits	2018-2023	111,000	
Mental Health online Survey (MHoS)	• Current and/or lifetime symptoms of common psychiatric disorders (depression, bipolar disorder, attention-deficit/hyperactivity disorder, eating disorders, generalized anxiety disorder, psychotic experiences, post-traumatic stress disorder, suicidal ideation and attempts, substance abuse, problematic gambling)<br>• Lifestyle (substance use, physical activity, screen time)<br>• Childhood psychosocial environment<br>• Stressful life events<br>• Social support<br>• Psychiatric medication effects and side effects	March-July 2021	86,000	https://doi.org/10.1093/ije/dyae017
Adverse events from medicines and vaccines (ADE-Q)	• Adverse events from medications<br>• Adverse events from vaccines<br>• Specification of medication and adverse event	April-September 2022	45,000	
Personality	Estonian NEO-PI-3	2008-2015	2,000	https://osf.io/97pvz/
	One Hundred Nuances of Personality (100NP)	November 2021 – March 2022	77,000	https://osf.io/97pvz/

* Anthropometry and objective measurements were determined by medical professionals.
** Anthropometry was filled by participants.

Supplementary Table 2. Omics datasets EstBB.

**OMICS profiling**	**N**
Genome-wide genotyping arrays	212,000
Metabolomics (NMR)	200,000
Whole genome sequencing	2,800
Whole exome sequencing	2,500
Microbiome oral, stool (gut metagenomics)	2,500
Telomere length	5,200
Clinical biochemistry	2,700
Metabolomics (MS/MS)	1,100
Metabolomics (LC-MS and Metabolon)	1,600
Genome-wide gene expression arrays	900
IgG glycosylation	1,000
Genome-wide methylation arrays	700
mRNA sequencing	600
Proteomics (SomaLogic)	600
Proteomics (Olink)	500

Supplementary Table 3. Frequencies of evaluated PGx phenotypes (and activity scores where available) in EstBB. The corresponding average frequencies for the European population are provided primarily based on PharmGKB gene-based frequency tables. A phenotype with a number (e.g. Intermediate Metabolizer 1.0) indicates the estimated activity score of this gene.

Gene	PGx phenotype	N in EstBB	EstBB frequency	European frequency
CYP2C19	Intermediate Metabolizer	47,518	22.5%	26.1%
CYP2C19	Normal Metabolizer	77,260	36.6%	39.6%
CYP2C19	Poor Metabolizer	3,526	1.7%	2.4%
CYP2C19	Rapid Metabolizer	64,606	30.6%	27.1%
CYP2C19	Ultrarapid Metabolizer	14,876	7.0%	4.6%
CYP2C9	Intermediate Metabolizer 1.0	26,057	12.3%	13.8%
CYP2C9	Intermediate Metabolizer 1.5	32,543	15.4%	20.8%
CYP2C9	Normal Metabolizer 2.0	148,967	70.5%	62.8%
CYP2C9	Poor Metabolizer 0.0	1,014	0.480%	0.6%
CYP2C9	Poor Metabolizer 0.5	2,676	1.3%	2.0%
CYP3A5	Intermediate Metabolizer	27,321	12.9%	13.7%
CYP3A5	Normal Metabolizer	1,064	0.504%	0.5%
CYP3A5	Poor Metabolizer	182,872	86.6%	85.7%
DPYD	Intermediate Metabolizer	12,275	5.8%	**3–7%
DPYD	Poor Metabolizer	59	0.028%	**0.3%
IFNL3	Favorable Response Genotype	91,490	43.3%	*38.0%
IFNL3	Unfavorable Response Genotype	119,767	56.7%	*63.0%
NUDT15	Intermediate Metabolizer	2,722	1.3%	0.8%
NUDT15	Normal Metabolizer	208,527	98.7%	98.6%
NUDT15	Poor Metabolizer	8	0.004%	0.001%
SLCO1B1	Decreased Function	72,912	34.5%	28.3%
SLCO1B1	Increased Function	3,504	1.7%	3.0%
SLCO1B1	Normal Function	122,698	58.1%	65.6%
SLCO1B1	Poor Function	10,748	5.1%	2.9%
TPMT	Intermediate Metabolizer	13,297	6.3%	8.4%
TPMT	Normal Metabolizer	197,744	93.6%	90.9%
TPMT	Poor Metabolizer	215	0.1%	0.2%
VKORC1	Normal Metabolizer	89,818	42.5%	58.7%
VKORC1	Intermediate Metabolizer	95,805	45.4%	*41.3%
VKORC1	Poor Metabolizer	25,634	12.1%	
All genes	At least 1 non-normal PGx Phenotype	211,241	99.99%	NA

*Based on the 1 variant carrier frequency in CPIC publication. **Based on the frequencies within the CPIC guideline publication.

Supplementary Table 4. Overview of data obtained via linking with national registries. Average rows per participant is calculated for the subset of participants who have data from the respective source (as indicated in the "% of all participants column").

Source	Participants	First record date	Last record date	% of all participants	Average rows per participant
Baseline questionnaire	158,705	2002/10	2024/04	74.8%	1.05
Self-reported diagnoses	50,809	1920/01	2018/01	23.9%	10.85
EHIF treatment bill	211,767	2001/08	2023/12	99.8%	121.24
EHIF diagnoses	211,767	2001/08	2023/12	99.8%	182.02
EHIF prescriptions	211,371	2004/01	2023/12	99.6%	150.21
eHealth medical case report	206,152	1998/01	2022/12	97.1%	38.16
Diagnoses from medical case report	206,109	1970/01	2022/12	97.1%	64.32
Cancer events	17,465	1955/08	2021/12	8.2%	1.17
Cancer screening events	114,304	2015/01	2021/12	53.9%	2.64
Medical Birth Registry	62,468	1992/01	2023/01	45% (of females)	1.92
Death certificates	10,723	2003/05	2024/04	5%	1
NEMC medical cases	114,098	1993/02	2020/11	53.7%	10.82
NEMC diagnoses	108,748	1993/02	2020/11	51.2%	14.99
TUH medical case	136,301	1999/12	2023/09	64.2%	16.89
TUH diagnoses	118,879	1999/12	2023/09	56%	27.02

EHIF – Estonian Health Insurance Fund, NEMC – North Estonian Medical Centre, TUH – Tartu University Hospital.

Supplementary Table 5. Thirty most common EHIF diagnoses present in EstBB.

ICD-10 main code	ICD-10 main diagnosis	N of EstBB participants	% of EstBB participants with diagnosis
J06	Acute upper respiratory infections of multiple and unspecified sites	166,111	78.3%
M54	Dorsalgia	137,896	65.0%
U07.1-U07.2	Emergency use for COVID-19	122,683	57.8%
H52	Disorders of refraction and accommodation	112,607	53.1%
B34	Viral infection of unspecified site	99,975	47.1%
J20	Acute bronchitis	92,492	43.6%
J02	Acute pharyngitis	89,552	42.2%
M25	Other joint disorders, not elsewhere classified	87,882	41.4%
J01	Acute sinusitis	79,774	37.6%
R10	Abdominal and pelvic pain	78,573	37.0%
U11	Need for immunization against COVID-19	77,848	36.7%
M79	Other soft tissue disorders, not elsewhere classified	76,336	36.0%
H10	Conjunctivitis	75,679	35.7%
N30	Cystitis	73,001	34.4%
J00	Acute nasopharyngitis [common cold]	70,577	33.3%
I10	Essential (primary) hypertension	68,079	32.1%
E78	Disorders of lipoprotein metabolism and other lipidaemias	66,737	31.4%
J04	Acute laryngitis and tracheitis	66,430	31.3%
J30	Vasomotor and allergic rhinitis	64,724	30.5%
B37	Candidiasis	62,396	29.4%
K29	Gastritis and duodenitis	61,985	29.2%
J03	Acute tonsillitis	61,786	29.1%
J35	Chronic diseases of tonsils and adenoids	58,845	27.7%
K21	Gastro-oesophageal reflux disease	57,014	26.9%
D22	Melanocytic naevinaevus	55,938	26.4%
N76	Other inflammation of vagina and vulva	55,814	26.3%
L23	Allergic contact dermatitis	53,668	25.3%
F32	Depressive episode	53,428	25.2%
B35	Dermatophytosis	52,175	24.6%
K04	Diseases of pulp and periapical tissues	52,148	24.6%

Supplementary Table 6. Twenty most common causes of death among EstBB participants.

ICD10 code	Diagnosis	N
I25	Chronic ischaemic heart disease	1,310
I11	Hypertensive heart disease	1,221
C34	Malignant neoplasm of bronchus and lung	440
I63	Cerebral infarction	360
I13	Hypertensive heart and renal disease	350
I21	Acute myocardial infarction	289
C25	Malignant neoplasm of pancreas	282
C16	Malignant neoplasm of stomach	225
C50	Malignant neoplasm of breast	218
C18	Malignant neoplasm of colon	209
U07.1-U07.2	Emergency use for COVID-19	193
C61	Malignant neoplasm of prostate	189
E11	Type 2 diabetes mellitus	157
I50	Heart failure	152
K70	Alcoholic liver disease	152
C71	Malignant neoplasm of brain	133
J44	Other chronic obstructive pulmonary disease	128
I42	Cardiomyopathy	124
X70	Intentional self-harm by hanging, strangulation, and suffocation	113
C22	Malignant neoplasm of liver and intrahepatic bile ducts	108

Supplementary Table 7. Characteristics of EstBB participants based on two recruitment waves.

Characteristic	Baseline Questionnaire 1	Baseline Questionnaire 2
Mean age (range)	44.3 (18-103)	42.6 (18-105)
Sex	65.6% women	67.9% women
**Nationality**		
Estonian	81.5%	93.7%
Russian	15.3%	4.8%
Other	3.2%	1.5%
**Education**		
Primary or basic	17.9%	4.7%
Secondary or secondary vocational	57.4%	44.4%
University degree	24.7%	50.9%
**BMI categories (%)**		
<18.5	2.4%	2.2%
[18.5–25)	44.2%	48.5%
[25.0–30)	31.2%	31.2%
≥30.0	22.2%	18.1%
**Smoking status**		
Current	28.6%	14.4%
Former	13.7%	31.4%
Never	57.7%	54.2%
